# Divergent Perceptual Processes on Cyberbullying Between Victims and Aggressors: Construction of Explanatory Models

**DOI:** 10.3389/fpsyg.2018.00396

**Published:** 2018-03-26

**Authors:** Inmaculada Fernández-Antelo, Isabel Cuadrado-Gordillo

**Affiliations:** Psychology and Anthropology, Faculty of Education, Universidad de Extremadura, Badajoz, Spain

**Keywords:** cyberbullying, aggressor, victims, perception modeling, intentionality

## Abstract

Understanding the causes of adolescents' aggressive behavior in and through technological means and resources requires a thorough analysis of the criteria that they consider to be identifying and defining cyberbullying and of the network of relationships established between the different criteria. The present study has aimed at making a foray into the attempt to understand the underlying structures and mechanisms that determine aggressors' and victims' perceptions of the cyberbullying phenomenon. The sample consisted of 2148 adolescents (49.1% girls; *SD* = 0.5) of ages from 12 to 16 (*M* = 13.9; *SD* = 1.2). The data collected through a validated questionnaire for this study whose dimensions were confirmed from the data extracted from the focus groups and a CFA of the victim and aggressor subsamples. The analysis of the data is completed with CFA and the construction of structural models. The results have shown the importance and interdependence of imbalance of power and intention to harm in the aggressors' perceptual structure. The criteria of anonymity and repetition are related to the asymmetry of power, giving greater prominence to this factor. In its perceptual structure, the criterion “social relationship” also appears, which indicates that the manifestations of cyberbullying are sometimes interpreted as patterns of behavior that have become massively extended among the adolescent population, and have become accepted as a normalized and harmless way of communicating with other adolescents. In the victims' perceptual structure the key factor is the intention to harm, closely linked to the asymmetry of power and publicity. Anonymity, revenge and repetition are also present in this structure, although its relationship with cyberbullying is indirect. These results allow to design more effective measures of prevention and intervention closely tailored to addressing directly the factors that are considered to be predictors of risk.

## Introduction

The lack of agreement when defining and delimiting the concept of cyberbullying has been generating increasingly pronounced controversy about the criteria that determine it (Slonje and Smith, 2013). To the existing discrepancies among researchers on the conceptualization of cyberbullying, we must add the different perspectives that adolescents have about this construct. In this sense, we can find that young people classify certain virtual aggressions as episodes of cyberbullying without becoming them. Or worse, it could be that manifestations of cyberbullying are interpreted as harmless behaviors. On the other hand, the influence exerted by the experiences of aggression or victimization experienced in the definition of cyberbullying is not yet sufficiently verified. Research on the perceptions that adolescents have of cyberbullying has found that the type of involvement with any given cyberbullying situation significantly influences which criteria the adolescent considers as defining this construct (Vandebosch and Van Cleemput, 2008; Dredge et al., 2014).

### Identifying criteria of cyberbullying

The principal achievement would seem to be general agreement on five criteria that distinguish cyberbullying from aggressive behavior in on-line contexts. These criteria may be summarized as: power imbalance, intent to cause another person social, or psychological harm, repetition of aggressive behavior, anonymity, and publicity (Thomas et al., 2015).

#### Repetition

Thomas et al. (2017) considered repetition of the aggressive behavior to be a defining criterion of cyberbullying, and they take it to exist when there is continuous sending of threatening or insulting messages through virtual forums, mobile telephony, etc. Other researchers, however, warn of the limited relevance of this criterion when defining and identifying cyberbullying episodes, arguing that a single aggression that spreads uncontrollably (virality) may cause recurring harm to the victim in a similar way to that produced if the behavior was performed continuously (Hutson, 2016). Although embarrassing private content may only have been sent to one recipient, it may be seen and then forwarded by others, not only increasing the durability of the harm (Pieschl et al., 2015) but also the perception of its seriousness (Schultze-Krumbholz et al., 2014; Wright et al., 2017).

#### Imbalance of power

In cyber scenario, the perception of power is linked to the relative mastery of ICT skills (Barlett et al., 2017b). Knowledge of these tools facilitates the access to and manipulation and dissemination of private material, as well as creating obstacles to identifying the aggressor (Casas et al., 2013). Nonetheless, a victim's mastery of ICT does not prevent them from being subjected to episodes of cyberbullying, so that the relevance of this criterion might be less than at first would seem apparent.

#### Intention to harm

The lack of face to face communication causes biases in interpreting the meaning of the message, and this in turn leads to frequent confusion about the intentionality of the person with whom they are interacting or exchanging messages. However, despite these difficulties in identifying the intentionality of cyber behavior, Crosslin and Golman (2014) note that adolescents consider the intention to harm to be a major factor for an episode of aggression to be cyberbullying.

#### Anonymity

Kowalski et al. (2012) argue that anonymity may encourage certain people to act in a way that they would never consider in real life. The perception of the impunity that identity concealment allows favors the adoption of ethically reprehensible behavior, including the perpetration of aggression and other types of cybercrime (Compton et al., 2014; Barlett et al., 2017a).

#### Publicity

Finally, the publicity criterion is defined as the open and uncontrolled spread of an aggressive behavior. For researchers such as Patchin and Hinduja (2010), it is one of the criteria with greatest presence in cyberbullying. For Nocentini et al. (2010), however, while they recognize the relevance of this criterion as reflecting the seriousness of the cyber abuse, it is not a factor that defines or delimits the cyberbullying construct. Sticca and Perren (2013) report that Swiss adolescents and Chen and Cheng (2016) that young Taiwanese attach particular importance to the public dimension of cyber abuse, and that they consider it to be a defining characteristic of cyberbullying, in turn, determining the seriousness of the harm.

### Combination and interaction of criteria as key indicators to identify cyberbullying

Many of the studies addressing this topic use small samples and resort to exploratory analyses to determine which are the criteria for adolescents that have a direct relationship with cyberbullying, which criteria have an indirect relationship, and which have no relationship. These data will contribute to constructing a preliminary theoretical structural model that allows one to understand youngsters' perception of the phenomenon of cyberbullying (Palladino et al., 2017). These studies have been conducted in countries with different cultures. But this does not mean that culture is the variable that has to be taken as responsible for the divergences that are found since, in today's globalized world, perceptions spread rapidly regardless of culture or ethnicity.

Specifically, Baas et al. (2013) focused on the criteria of intentionality and repetition, and concluded that the perception which children aged 11–12 had of these criteria is ambiguous and arbitrary, and that therefore it is impossible to determine whether the relationship between these two criteria is the most used when defining cyberbullying. Instead, Menesini et al. (2012) note that many European adolescents understand that if a behavior is repeated then it cannot be classified as unintentional, which would reflect a relationship between the repetition and the intentionality criteria.

The relevance of certain criteria over was studied by Nocentini et al. (2010). They noted the importance European adolescents give to such criteria as intentionality and imbalance when differentiating an act of cyber aggression from a cyberbullying episode. However, Dredge et al. (2014) found that very few Australian adolescents take intention to harm and imbalance of power to be essential components in the definition of cyberbullying.

Results concerning the perception of other criteria, such as anonymity or giving publicity to the aggression, are sparse and contradictory. Schultze-Krumbholz et al. (2014) affirm that adolescents recognize the influence of anonymity and publicity on the seriousness of the cyber aggression but that they do not consider these two factors to be defining characteristics of cyberbullying.

Cuadrado and Fernández (2016) show that adolescents' perception of cyberbullying depends on the role they play of aggressor or victim: victims consider that intentionality, publicity, and imbalance of power are directly related to cyberbullying, with intentionality having the greatest influence of the three; aggressors put power imbalance as being the most important dimension defining cyberbullying, followed by the intention to hurt. Finally, studies such as those of Betts and Spenser (2017) note the normalization of violent behavior as patterns of social relationships and interaction among adolescents. This distorted perception may be a predictor of cyberbullying (Cuadrado and Fernández, 2016). These results, together with those of the works mentioned above, allow us to approach the construction of a preliminary theoretical structural model of adolescents' perception of cyberbullying.

### The study

The numerous controversies and contradictions that still exist regarding the delimitation of the cyberbullying construct demonstrate the need for further research focused on determining the criteria that shape the structure of the perceptions that adolescents have of this phenomenon and on seeking explanations of this behavior. Previous studies analyzed the dependency relationships between pairs of criteria identifying cyberbullying, especially between imbalance and intentionality. However, to understand the underlying mechanisms that define the perception adolescents have of this construct it is not enough to analyse pairs of criteria. It is also necessary to examine the web of potential relationships that includes all the possible factors, both directly observable and latent, that may be attributed to the cyberbullying construct. In the case of the victims, the review of the scientific literature indicates that the continued experimentation of the damage caused by cyber attacks (repetition criterion) reinforces the perception of intentionality of the aggressor to cause psychological, emotional, social damage, etc. In this way, the repetition criterion could maintain an indirect relationship with cyberbullying exerted through the intentionality criterion. Likewise, the absence of technical knowledge to reveal the identity of the aggressors would place this technological domain (power imbalance) as a criterion of first order and the anonymity criterion dependent on it. In addition, in the case of aggressors, the normalization of humiliating behaviors as patterns of relationship between adolescents could cause the criteria of revenge and advertising to maintain an indirect relationship with the cyberbullying construct mediated through these maladjusted social relationships with peers. The objectives of the present study were to: (i) construct possible explanatory models of the perception of cyberbullying from identifying and relating the criteria that form this construct; (ii) determine the predictive values of the criteria of repetition, imbalance, intentionality, publicity, anonymity, revenge, and social relationships for adolescents' perception of cyberbullying; and (iii) analyse the influence of previous cyber victimization and cyber aggression experiences in the construction of explanatory models of the perception of cyberbullying.

## Methods

### Sample

The sample consisted of 2,148 adolescents (50.9% boys and 49.1% girls; *SD* = 0.5) of ages from 12 to 16 (*M* = 13.9; *SD* = 1.2).

To select the participants, we applied a stratified multistage, approximately proportional, sampling procedure with conglomerates and random selection of groups in public secondary schools in which Compulsory Secondary Education (ESO) is taught. The strata considered were the provinces and geographical areas of Extremadura (Spain), selecting towns in the north, south, east, and west of the region, and taking their different socio-cultural contexts into account. The conglomerates used were the secondary schools. In each school, one of the four courses making up the ESO (1st year, ages 12–13; 2nd year, age 14; 3rd year, age 15; and 4th year, age 16) was selected at random.

### Questionnaire design

The instrument used for the collection of data was a questionnaire of 28 questions grouped into nine blocks. The first block consists of three questions that allow one to identify whether the adolescents consider themselves to be aggressors, victims, or witnesses of cyberbullying. From this identification, we can analyse how they behave in the rest of the questionnaire, i.e., what perception they have of the phenomenon of cyberbullying. These first three questions also provide insight into how often during the last 3 months they had committed, been victims of, or observed cyberbullying episodes. The scale used comprised four values: “never,” “once or twice,” “once a week,” and “several times a week.” This scale has been used in many studies analyzing the prevalence of cyberbullying (e.g., Hemphill et al., 2012; Huang and Chou, 2013; Del Rey et al., 2015). A respondent is considered to have played the role of aggressor, victim, or witness when they say they have been involved at least 1 or 2 times in some of the behaviors they are presented with. It is important to note that the adolescents who manifested themselves as having been both victims and aggressors in any of the modalities that will be presented below were excluded from both the aggressor and victim subsamples, since they play some other role such as bully/victim which we do not analyse in the present study. In the following, we present by way of example the question that allows the adolescents who consider themselves to be victims of cyberbullying to be identified. They were told to indicate how often during the past 3 months they had suffered any of the following behaviors: “(1) I have been insulted through the mobile phone or Internet; (2) I have been threatened or blackmailed through the mobile phone or Internet; (3) lies and false rumors have been spread about me through the mobile phone or Internet; (4) I have been removed from contact lists on social networks, group chats, or emails so as to exclude me; (5) I have had someone pretend to be me, and my email, private chat rooms, or social network profile have been accessed without my permission; (6) they have sent by mobile phone or Internet incriminating photos or videos, which are denigrating or demeaning to me; (7) they have recorded fights in which I participated and spread them through mobile phones, social networks, or other cyber means; (8) they have sent sexual or erotic type of content in which I took part.” If an adolescent answers one or more of these items indicating a frequency of “at least once or twice” and does not declare having committed any of these abuses, they are assigned to the victim subsample. The aggressor subsample is identified analogously.

A reliability analysis of the instrument showed satisfactory internal consistency of the blocks of items aimed at identifying the aggressors, victims, and witnesses (Cronbach's alpha: α = 0.87; α_aggression_ = 0.84; α_victimization_ = 0.90; α_witnesses_ = 0.77).

In addition to an exhaustive review of the scientific literature on the topic, focus group sessions with a sample of 49 adolescents (16–18 years) grouped into teams of 7 were employed in preparing the questions directed at determining adolescents' perception of cyberbullying. In these sessions the adolescents were given 15 descriptions of different kinds of cyber attacks and were asked to interpret them, analyzing the goal of the aggressor, the possible reasons that led him or her to commit that certain type of abuse, as well as the implications for the victim. From the explanations that were given for each of the descriptions presented, we categorized the responses in accordance with the absence or presence of the criteria that adolescents associate with the conceptualization of cyberbullying. Based on these responses, a principal component analysis was carried out to extract the criteria that explain a large part of the total variability. The result of this analysis led to seven criteria being selected (Table 1): the aggressor's intention to cause harm (Component 1), imbalance of power between the aggressor and victim (Component 2), publicity made of the aggression (Component 3), social relationships and forms of communication used by adolescents in the cyber world (Component 4), repetition of the abuse (Component 5), anonymity behind which those who abuse others hide (Component 6), and revenge (Component 7), whose Cronbach's alpha reliability values ranged between 0.71 and 0.83.

**Table 1:** Total variance explained by the components.

	**Initial eigenvalues**			**Sum of the squared saturations of the extraction**		
**Component**	**Total**	**% of variance**	**Accumulated %**	**Total**	**% of variance**	**Accumulated %**
1	5.13	21.18	21.18	5.13	21.18	21.18
2	4.68	19.32	40.50	4.68	19.32	40.50
3	4.03	16.64	57.14	4.03	16.64	57.14
4	2.95	12.18	69.32	2.95	12.18	69.32
5	2.07	8.55	77.87	2.07	8.55	77.87
6	1.74	7.18	85.05	1.74	7.18	85.05
7	1.23	5.08	90.13	1.23	5.08	90.13
8	0.87	3.59	93.72			
9	0.51	2.11	95.83			
10	0.48	1.98	97.81			
11	0.32	1.32	99.13			
12	0.21	0.87	100			

*Extraction method: principal component analysis*.

These criteria were incorporated in the form of items into the questionnaire's 25 remaining questions aimed at determining the perception of cyberbullying and the modalities in which it manifests itself. The 25 questions are grouped into 8 thematic blocks corresponding to the different modes in which this phenomenon manifests itself in accordance with the “type of behavior” criterion: insults (including homophobia), threats (including blackmail), spreading false rumors, exclusion (from contact lists, social networking, etc.), identity theft, sexting, posting denigrating images or videos, and recording and disseminating physical aggressions (Willard, 2006; Huang and Chou, 2010; Rivers and Noret, 2010; Kowalski et al., 2012). Each but one of these blocks comprises 3 questions. The exception is the “insults” mode for which there are 4 questions to try to cover the great variety of types of insults that were encountered. With these questions, we can determine the perception adolescents have of behaviors regarded as manifestations of cyberbullying, and the criteria they use to define those behaviors. The scale comprises 5 values to indicate the degree of agreement with each of the items presented (strongly agree, agree, neither agree nor disagree, somewhat disagree, and disagree). Multi-item measurements help to minimize the perceptual bias of the respondent (Selkie et al., 2015). Authors such as Asún et al. (2016) consider that a variable can be treated as a (continuous) scale when its values represent ordered categories with a metric with meaning. These authors affirm that in studies of Social Sciences and Psychology, it would be possible to consider the ordinal variables as continuous variables, understanding as values the cut-off points of the continuous variable.

Once a draft had been prepared of the 25 questions, it was presented to a group of 78 adolescents to determine the questions' reliability and the degree of comprehension and familiarity with the terms used in the questions. A reliability analysis showed satisfactory internal consistency in the block of items designed to access the perceptions of cyberbullying (Cronbach's α = 0.79). We also calculated the degree of internal consistency for each of these eight thematic blocks. The following are the results: insults (α = 0.82), threats (α = 0.71), spreading false rumors (α = 0.76), exclusion (α = 0.78), identity theft (α = 0.85), sexting (α = 0.79), posting denigrating images or videos (α = 0.77), and recording and disseminating physical aggressions (α = 0.82). Subsequently, once the data of the total sample had been input, the internal consistency coefficients were recalculated. The results did not vary significantly. The following are some examples of the questions included in these thematic blocks.

An example of this type of questions is: “Why do you think some peers threaten others through telephone calls? (1) Because they do not dare do it face to face for fear of reprisals; (2) Because they can hide their identify and inflict fear on others who are stronger; (3) Because it is the way they have of relating; (4) Because that way they feel more powerful; (5) Because it the only way they have to get what they want; (6) Because they feel more accepted by their friends; (7) Because it is a way of getting revenge; (8) Because they record the telephone calls and then spread them so that the victim repeatedly feels fear; (9) Because they like to see how others suffer; (10) They are jokes or other ways of having fun that are typical of adolescents.”

Another example would be: “When a peer continuously insults another person through the mobile phone or the Internet, I consider this conduct to be… (1) something normal among adolescents; (2) the usual way we have of relating; (3) harmless behavior if it occurs sporadically; (4) something irrelevant if the person who insults me is not important to me or unknown; (5) harmless behavior if it occurs in private; (6) an aggression when it harms another person; (7) a form of revenge against others whom you do not like or who have attacked you; (8) an aggression if done by a popular person; (9) an aggression if the insult is accompanied by an offensive image.”

Subsequently, a confirmatory factor analysis was performed of the victim and aggressor subsamples in order to confirm the dimensions of the questionnaire when applied to particular groups.

### Procedure

With this being a study involving minors, it was necessary to have the parents' consent, and the approval of the Regional Administration's education inspectors and of the different schools' management teams.

To obtain the parents' consent, they were sent a letter describing the nature of the study, the use that would be made of the data, and the commitment to confidentiality and anonymity. This letter was accompanied by a form for the parents to forward to the school if they did not want their children to participate in the study.

The education inspectors and management teams were sent a report in which the objectives of the research, the procedures, and the guarantee of anonymity of the participants were detailed. This was thus in full compliance with the ethical standards governing secondary schools. Previously, both the research objectives and the procedure, instruments and techniques used were supervised and approved by the Ethics Committee of University of Extremadura (Spain).

The data acquisition procedure followed once the parents and school authorities had been informed consisted in the researchers going to each of the selected schools in turn, where they distributed the questionnaires in each of the classes, and remained in those classrooms until all of the participants who had voluntarily wanted to take part had handed them back filled in. For focus groups, only participants (16–18 years) whose parents had given informed consent were selected. This consent document explains to the parents the activity that their sons and daughters are going to carry out, what use will be made of the information collected and the guarantees of anonymity that we offer.

### Data analysis

In accordance with the objectives outlined in this paper, we created different structural models that tested previously by confirmatory factor analyses. These analyses were carried out on: (i) the cyber victim subsample, and (ii) the cyber aggressor subsample. The resulting structural equation models were subjected to maximum likelihood estimation. To check their fit, we used the chi-squared statistic, the comparative fit index (CFI), the goodness-of-fit index (GFI), the Tucker-Lewis index (TLI), the root mean square error of approximation (RMSEA), and the root mean residual (RMR). To check for overfit in the resulting models, we applied measures of parsimony fitting: the parsimonious normed fit index (PNFI) and the parsimony goodness-of-fit index (PGFI). We also estimated the standardized regression coefficients included in the models.

## Results

### The cyber victim subsample

The results yielded a cyber victim subsample of 328 participants (131 boys and 197 girls) who claimed to have been subjected to cyber or mobile phone aggression by their peers in the past 3 months. Those who identified themselves as both victims and aggressors were excluded from the study as playing roles that could be likened to that of the bully-victim or victim-aggressor, thus diverging from the objectives of the present work.

The confirmatory factor analysis of the dimensions that comprise the victims' perceptions of cyber aggression showed an adequate fit of the factorial solution: χ^2^*/df* = 1.064, *p* < 0.01; RMSEA = 0.043; RMR = 0.031; CFI = 0.968; TLI = 0.953; GFI = 0.952.

The correlation analysis showed positive direct influences of imbalance (*r* = 0.31, *p* < 0.05), intentionality (*r* = 0.69, *p* < 0.001), publicity (*r* = 0.41, *p* < 0.01), and revenge (*r* = 0.27, *p* < 0.05) on the cyberbullying variable, and a negative direct influence of social relationship (*r* = −0.51, *p* < 0.01) (Table 2).

**Table 2:** Correlations between the variables that form the victims' perception of cyberbullying behavior.

	**1**	**2**	**3**	**4**	**5**	**6**	**7**	**8**
Intentionality								
Imbalance	0.44^**^							
Publicity	0.62^***^	0.21^*^						
Anonymity	0.29^*^	0.56^***^	−0.18					
Repetition	0.47^**^	0.09	0.75^***^	0.13				
Revenge	0.50^**^	0.26^*^	−0.03	−0.12	0.07			
Social Relationship	−0.53^**^	−0.21^*^	0.38^**^	−0.26^*^	0.29^*^	−0.32^**^		
Cyberbullying	0.69^***^	0.31^*^	0.41^**^	0.17	0.20	0.27^*^	−0.51^**^	

* p < 0.05;

** p < 0.01;

*** *p < 0.001*.

**Figure 1:**
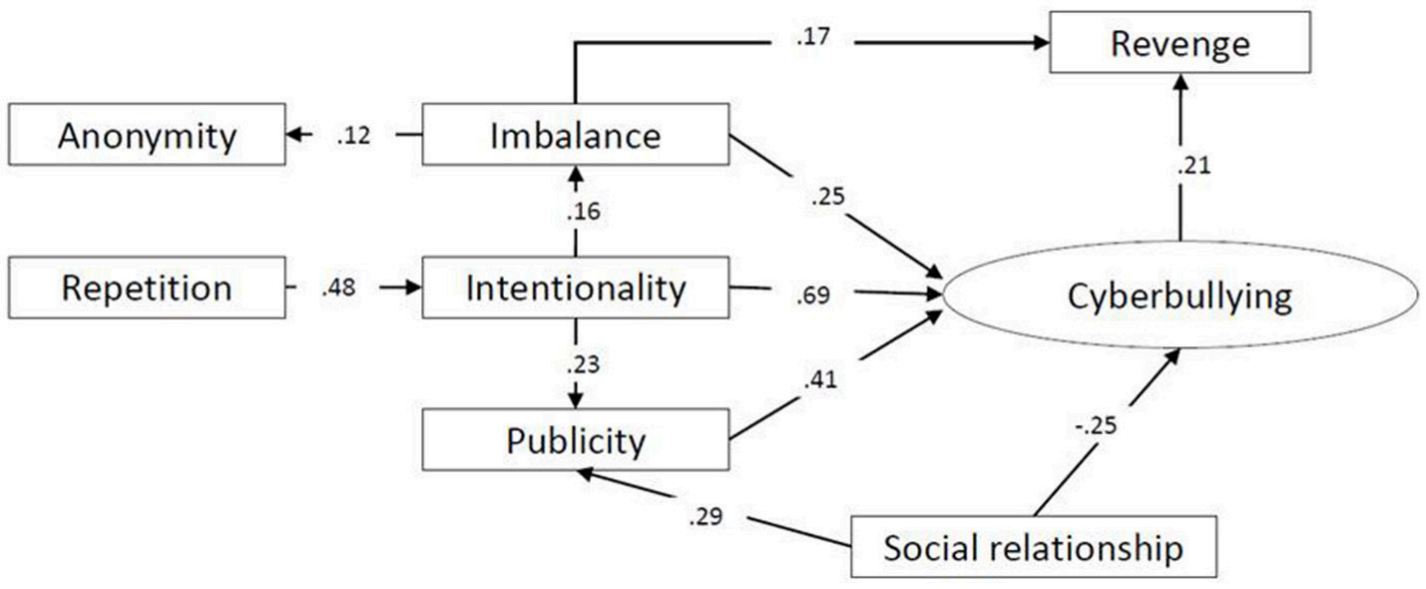
Structural equation model of the cyber victim.

The structural equation model that emerged from the analysis of the cyber victim data comprised seven standardized observable variables and one latent variable, cyberbullying (Figure 1). The calculated fitting indices showed the fit of the model to be correct: χ^2^ = 19.425; χ^2^*/df* = 1.284, *p* = 0.136; RMSEA = 0.033; RMR = 0.001; CFI = 0.976; TLI = 0.987; GFI = 0.981; NFI = 0.980.

The calculated parsimony fit indices allow us to affirm that the resulting model is not overfitted: PGFI = 0.56; PNFI = 0.64.

The standardized regression coefficients showed a positive predictive relationship of the cyberbullying variable with the variables intentionality (β = 0.691, *p* < 0.001), imbalance (β = 0.248, *p* < 0.01), and publicity (β = 0.409, *p* < 0.01), and a negative relationship with the social relationship variable (β = −0.252, *p* < 0.01). This last variable also and in turn predicts the publicity given to the cyber aggression (β = 0.290, *p* < 0.05).

It is important to note that the model also indicates that the victims associate cyberbullying with revenge (β = 0.167, *p* < 0.05), and that the influence of repetition on the cyberbullying variable is not direct but indirect through the intentionality variable (β = 0.476, *p* < 0.05). Finally, concerning the variable anonymity, the victims predict its existence through the imbalance of power (β = 0.118, *p* < 0.05), although they do not believe that anonymity is a predictor of cyberbullying.

The relationships between the variables in this model explain 53% of the variance of the cyberbullying variable.

### The cyber aggressor subsample

The cyber aggressor subsample consisted of 380 participants (232 boys and 148 girls) who reported having carried out cyber or telephone abuse with the intent to harm some of their peers during the last 2 months.

The confirmatory factor analysis of the dimensions that comprise the aggressors' perceptions of cyber aggression showed a correct fit of the factorial solution: χ^2^*/df* = 1.425, *p* < 0.01; RMSEA = 0.039; RMR = 0.028; CFI = 0.975; TLI = 0.962; GFI = 0.971.

**Figure 2:**
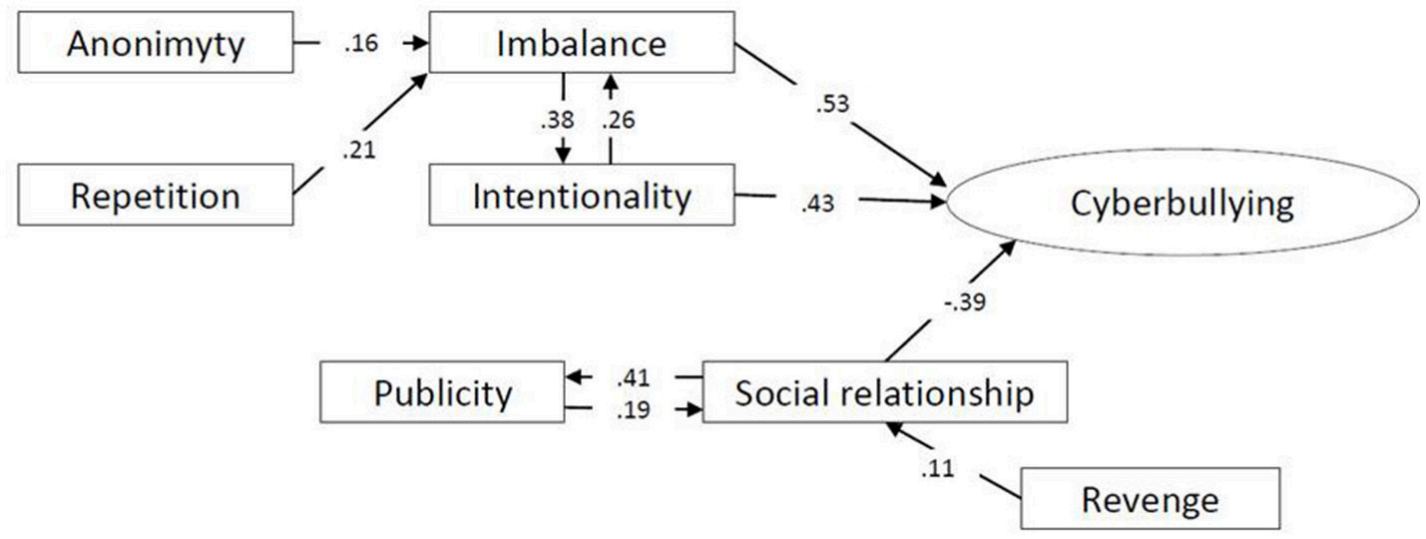
Structural equation model of the cyber aggressor.

The correlation analysis showed the direct influence on the perception of cyberbullying of three variables, two of them positive – imbalance (*r* = 0.45, *p* < 0.01) and intentionality (*r* = 0.27, *p* < 0.05)—and one negative—social relationship (*r* = −0.19, *p* < 0.05). Based on these results and on the correlations found between the dimensions that configure the perception of cyber aggression, a structural equation model was constructed consisting of seven standardized observable variables and one latent variable, cyberbullying (Figure 2). The calculated fitting indices showed a correct fit of the model: χ^2^ = 19.425; χ^2^*/df* = 1.521, *p* = 0.186; RMSEA = 0.042; RMR = 0.018; CFI = 0.970; TLI = 0.977; GFI = 0.974; NFI = 0.969.

The calculated parsimony fit indices allow us to affirm that the resulting model is not overfitted: PGFI = 0.58; PNFI = 0.67.

The standardized regression coefficients reflected in this model indicate that there are two variables (anonymity and repetition) which predict the imbalance of power between aggressor and victim, and, in turn, this asymmetry of power strongly predicts the perception of cyberbullying (β = 0.548, *p* < 0.01). Also, a relationship of interdependence can be observed between the variables imbalance and intentionality, both predictive of cyberbullying. The social relationship variable, closely linked to publicity, is a negative predictor of cyberbullying (β = −0.437, *p* < 0.01). Finally, there stands out the link between revenge and social relationship (β = 0.092,*p* < 0.05).

## Discussion

In a technological society like today, in which 97% of adolescents between the ages of 12 and 18 use social networks to communicate, share information of all kinds, socialize, or simply have fun (Garmendia et al., 2011), new forms and codes of power emerge, new ways of managing emotional states, of making decisions about friendships, etc. In this scenario, in which what is virtual is occupying an increasingly prominent place and in which the rules of interaction that are prevalent in cyberspace are not always compatible with those established in the physical world, new forms of conflict and violence appear to be caused by the misinterpretation of those rules, or by deliberately ignoring them (Udris, 2014).

The results presented in this paper have revealed the web of interactions that adolescents establish between the criteria that configure their perceptions of cyberbullying. As against the five criteria (intentionality, imbalance, repetition, publicity, and anonymity) that many researchers set as key factors in identifying this phenomenon (Kowalski et al., 2012), the perceptual model of these Spanish adolescents showed that just three of these criteria have a direct influence on their definition of cyberbullying: intentionality, imbalance, and publicity.

Thus, in this model, repetition of the aggressive behavior is excluded from being a determinant factor, to become a factor that is secondarily associated with the publicity criterion. The little relevance that adolescents attach to the repetition of cyber abuse can be explained by the characteristics of the new means and forms of communication that prevail in cyberspace. Speed and the lack of control over the spread of the aggressive behavior once it has been posted to social networks or communicated by mobile phone may, as indicated by Mishna et al. (2010), result in reiterated harm to the victim even though the abuse as such only occurred once.

Another criterion that adolescents relegate to the background is anonymity. Despite the results of some other studies indicating that young people consider the hiding of the aggressor's identity to be predictive of cyberbullying (Hoff and Mitchell, 2009; Udris, 2014), the knowledge or reasonable suspicions that many victims and witnesses have of the authorship of the abuse would explain why this criterion is relativized and linked secondarily to the imbalance of power, understood this latter as skill with the use of technological resources to hide the aggressor's identity.

In addition to these five criteria and their interrelationships, Compton et al. (2014) indicated that, for some young people, fun or entertainment may be constitutive or predictive factors of cyberbullying. However, in the present study we found that adolescents legitimize some cyber aggression by alluding to the emergence of new forms of interaction and communication characteristic of their generation, and therefore do not classify these behaviors as being episodes of cyberbullying. The selective application of moral standards would explain how the same abuse can be interpreted at times as a way of having fun and at other times as being deliberate aggression. This controversial form of dual reasoning may be motivated either by the detection of low levels of ethical competence (Müller et al., 2014) or by an attempt to avoid feeling guilty or accepting certain responsibilities (Sticca and Perren, 2015). The negative relationship that the adolescents in this study established between the social relationship criterion and the cyberbullying construct is evidence for the existence of certain imbalances in their moral reasoning.

Finally, in the explanatory model of the perceptions of cyberbullying constructed from the results of this study there emerges a new factor: revenge. Although Crosslin and Golman (2014) suggested that American adolescents understand revenge to be a motive or reason for the appearance of cyberbullying, Spanish adolescents see revenge to be a justifiable reaction of the victims to cyberbullying experiences that they have suffered.

But undoubtedly one of the factors that has the greatest influence on the determination of the perceptions of cyberbullying is that of previous cyber aggression and cyber victimization experiences. This is evidenced in the present study by the major differences between the explanatory models of the aggressors' and the victims' perceptions of cyberbullying. Although both aggressors and victims coincide in pointing to imbalance and intentionality as predictors of cyberbullying, the aggressors emphasize imbalance whereas the victims emphasize intentionality. The aggressors' possible lack of awareness or inability to foresee the effects that their actions or offensive comments will have on their peers could lead to the aggression committed not being perceived as a moral transgression. As a result, they do not attribute the intention to harm to these acts (Talwar et al., 2014). It is also possible, as noted by Staude-Müller et al. (2012), that the adolescent has internalized and normalized abusive behavior as being seemingly harmless patterns of social relationships with their peers, and therefore they not only do not perceive any intention to harm, but they also establish a strong antagonistic link between social relationship and cyberbullying. For the victims, the allocation of less importance to the imbalance criterion may, according to Park et al. (2014), be because of the perception they have of the type of relationship between aggressor and victim, in which there is not always any confirmation of an asymmetry of power.

The aggressor and victim explanatory models of the perceptions of cyberbullying also differ in how the publicity criterion is considered. The aggressors closely link this criterion to mechanisms of social interaction, whereas the victims conceive it to be predictive of cyberbullying. The repeated experience of the harm suffered as a result of the dissemination and publicity of the abuse that they have suffered may explain why victims include this criterion as a key factor in the determination of cyberbullying.

Other differences between aggressors and victims are found in their perception of the anonymity criterion. Cyber aggressors perceived anonymity to be an action that contributes to increasing the imbalance of power. The victims, however, believe anonymity to be an obvious result of that same imbalance of power. Only those who have an advanced mastery of ICT skills can effectively make themselves anonymous.

Regarding the differences concerning the repetition of aggressive behavior criterion, the cyber aggressors consider repetition of the abuse to be an explicit manifestation of ostentation of power. The search for social recognition and acceptance by their peers generates in these adolescents the need to continuously display their power, even if they have to resort to ethically reprehensible behavior. The victims, however, understand repetition to be a clear sign of the intention to cause harm. As noted by Menesini et al. (2012), if an abuse occurs repeatedly it cannot be understood to be a fortuitous harmless act, but as a deliberate action that seeks to harm others.

Finally, we detected important differences in the interpretation that aggressors and victims make of the revenge criterion. Those who carry out abuse against their peers conceived of revenge behavior as a mechanism of social interaction lacking any implied intention to harm. This would seem to show that the aggressors are less demanding in the moral evaluations of their behavior, and, as indicated by Talwar et al. (2014), they could be in a position of risking moral maladjustment, with an increased likelihood of interpreting aggressive and revengeful situations as fun or entertainment. On the contrary, the victims are convinced that being cyber-abused provokes a feeling of revenge that in part is related to a prior existence of an imbalance of power. From these results, and in accordance with König et al. (2010) and Runions (2013), for the victims, revenge or cyber revenge could represent a way of restoring the power balance and an increased sense of control and security. However, one must not forget that the feeling of revenge does not arise in a pure and isolated form, but is instead colored by other feelings and emotions that generate disproportionate reactions to the suffering that has been undergone.

## Conclusions

The search for explanations of aggressive and cyber aggressive behavior of adolescents is a recurring theme in psychological research. Nevertheless, despite the effort that has been made and the diversity of approaches taken, many questions remain. The present study has aimed at making a foray into the attempt to understand the underlying structures and mechanisms that determine aggressors' and victims' perceptions of the cyberbullying phenomenon. This phenomenon, though relatively new, constitutes a serious public health problem that affects children, adolescents, and even adults. The consequences of these problems are not virtual, but really and directly affect the population either through symptoms that may be internal (anxiety, sadness, depression, fear, insomnia,…) or external (behavioral problems, hyperactivity, delinquency), or through the emergence of new psychological and somatic symptoms symptoms of uncertain etiology (Aboujaoude et al., 2015).

The results have shown that previous cyber victimization and cyber aggression experiences lead to major differences in the explanatory models that adolescents construct to interpret cyber abusive behavior either as cyberbullying episodes, or as social relationship mechanisms, or as a revenge reaction to aggression that has been suffered.

In this regard, we note that the aggressors' explanatory model is based primarily on two factors: imbalance of power over the victim, and intention to harm. There was also found to be a strong reciprocal relationship between the two factors, demonstrating the importance and interdependence of these criteria in the aggressors' perceptual structure. The asymmetry of power takes on greater prominence, however, when one takes into consideration that it functions as a link promoting indirect causal relationships of the anonymity and repetition factors with the cyberbullying construct.

The victims' perceptual structure is based around three criteria: imbalance of power, intentionality, and publicity. But, unlike the aggressors, the key factor in this structure is not the asymmetry of power, but the intention to harm. This factor, in addition to maintaining a strong causal relationship with cyberbullying, can explain the existence and relevance of other criteria such as the imbalance of power or publicity in these adolescents' perception of cyber abuse. Finally, its status as a key element is further confirmed by the indirect relationship that it mediates between repetition and cyberbullying.

Another of the divergences found in the possible explanatory models of the aggressors' and victims' perceptions lies in the interpretations they make of the social relationship factor. Those who have occasionally committed cyber abuse try to legitimize the aggressiveness in their patterns of social interaction by alluding to a previously experienced feeling of revenge. At other times, these violent forms of relating are interpreted as patterns of behavior that have become massively extended among the adolescent population, and have become accepted as a normalized and harmless way of communicating with other adolescents over the network and by means of other technological resources.

But when victims justify the violent facet of their cyber interactions and do not classify them as abusive situations, they usually resort to explanations related to the attribution of a more playful and fun character than they get in their face-to-face interactions. Nonetheless, they note that when these types of relationships become massively extended then they may indirectly be the cause of cyberbullying situations.

With these structures of direct and indirect interactions between observable and latent factors, one can construct possible explanatory models that may help one understand the perceptions that aggressors and victims have of cyberbullying. It may then be possible to design more effective measures of prevention and intervention closely tailored to addressing directly the factors that are considered to be predictors of risk.

## Limitations

One limitation of the present study lies in the composition of the sample. The cluster used allowed adolescents in both rural and urban areas, comprising diverse socio-cultural contexts, to be included. But it took account of neither the availability of technological resources nor the participants' level of ICT competence. It would be interesting to consider these variables in future research, especially if two groups, such as aggressors and victims, are compared. Greater technological competence on the part of one of the groups could lead to a reorientation of how some results are interpreted.

## Author contributions

IF-A and IC-G are responsible for all tasks related to the design and development of the article, as well as the capture and analysis of the analyzed data.

### Conflict of interest statement

The authors declare that the research was conducted in the absence of any commercial or financial relationships that could be construed as a potential conflict of interest.
